# More than meets the IR: the expanding roles of variant Ionotropic Glutamate Receptors in sensing odor, taste, temperature and moisture

**DOI:** 10.12688/f1000research.12013.1

**Published:** 2017-09-26

**Authors:** Lena van Giesen, Paul A. Garrity

**Affiliations:** 1 National Center for Behavioral Genomics and Volen Center for Complex Systems Department of Biology, Brandeis University, Waltham, Massachusetts, USA

**Keywords:** ionotropic receptors, sensory perception, evolutionary genetics

## Abstract

The ionotropic receptors (IRs) are a branch of the ionotropic glutamate receptor family and serve as important mediators of sensory transduction in invertebrates. Recent work shows that, though initially studied as olfactory receptors, the IRs also mediate the detection of taste, temperature, and humidity. Here, we summarize recent insights into IR evolution and its potential ecological significance as well as recent advances in our understanding of how IRs contribute to diverse sensory modalities.

## Introduction

Identified in 2009 as a novel branch of the ionotropic glutamate receptor (iGluR) family ^1^, the ionotropic receptors (IRs) are emerging as important mediators of sensory transduction in invertebrates ^2, 3^. They were initially studied as receptors for volatile chemicals, often acids or amines ^1, 4– 6^, but recent work has greatly expanded our appreciation of their functional range. IRs have been found to detect other classes of chemicals and to mediate modalities beyond olfaction, including gustation, thermo-sensation, and humidity sensation (hygro-sensation) ^7– 14^. The diversification of IR function across species has also made IRs excellent subjects for investigating the evolution of sensory perception. In this review, we summarize current views of IR function, emphasizing recent advances in understanding the contribution of this receptor family to many aspects of sensory biology.

## The functional organization of the ionotropic receptor subfamily of ionotropic glutamate receptors

The iGluRs are a large and ancient gene family, present in genomes from plants to animals. Among animal iGluRs, IRs form an invertebrate-specific subfamily that has a common ancestor with AMPA (α-amino-3-hydroxy-5-methyl-4-isoxazolepropionic acid) and Kainate receptors, postdating their collective divergence from NMDA ( *N*-methyl D-aspartate) receptors ^2, 15^. IRs are functionally distinct from other classes of animal iGluRs: whereas NMDA, AMPA, and Kainate receptors are widely expressed in the nervous systems and mediate excitatory synaptic transmission in response to the amino acid glutamate ^16^, IRs are predominantly expressed by sensory neurons and act as receptors for diverse sensory stimuli. iGluRs form tetrameric cation channels, sometimes as homo- and sometimes as hetero-tetramers ^16^, and the potential to form mixed oligomers appears fundamental to IR signaling. In many cases, broadly expressed “co-receptor” IRs, like IR25a or IR8a, form heteromeric partnerships with stimulus-specific IRs, generating an array of receptors with diverse specificities ^3, 5^.

At the protein level, IRs resemble other iGluRs in possessing an extracellular ligand-binding domain and a transmembrane domain with three membrane-spanning and one pore region. NMDA, AMPA, and Kainate receptors also contain an amino-terminal domain (ATD) involved in receptor assembly, trafficking, and function ^16^. However, only a small subset of IRs possess ATDs. Of the 66 *Drosophila melanogaster* IRs, only the co-receptors IR25a and IR8a contain obvious ATDs, and a few other IRs (like IR21a, IR40a, and IR93a) contain appropriately sized regions (about 400 amino acids) that could act as ATDs, although they have limited amino-acid similarity to ATDs ^2^. In contrast, most IRs simply lack the sequences needed to form canonical ATDs (for example, in IR76b, this region is only about 150 amino acids), making it unclear how they form functional oligomers. Interestingly, many of the IRs that lack ATDs are “stimulus-specific” and require co-receptor IRs to function ^3, 5^. This suggests that co-receptor ATDs supply activities essential for the receptor complex to operate. Although this is an appealing paradigm, the extent to which it applies to all IRs is not yet clear, as emphasized by the proposed ability (discussed below) of the ATD-less IR76b to function without the assistance of IR25a or IR8a.

## New roles for ionotropic receptors in taste and smell

Although initial studies of IR function focused on their involvement in olfaction, many IRs are expressed in taste-sensing tissues of the larva and the adult fly, including the proboscis, pharynx, and legs, suggesting that IRs also contribute to gustation ^11, 13^. Although some IRs provide chemical specificities that appear to complement those provided by the gustatory receptor (GR) family of sensory receptors, important for the detection of many tastants ^9, 14^, recent work indicates that other IRs mediate the detection of chemicals that are also recognized by GR-expressing neurons ^17^. Sucrose strongly activates GR-expressing sweet receptors that elicit a robust appetitive response ^18– 20^. However, sucrose is also detected by IR60b-expressing gustatory neurons in the adult pharynx ^17^. In contrast to the appetitive effects of activating the GR-expressing sucrose sensors, activation of the IR-expressing sucrose sensors had the opposite effect, inhibiting feeding ^17^. In this way, IR60b-expressing gustatory receptor neurons (GRNs) can control sucrose consumption at the level of gustatory neuron function. It is interesting to consider whether the balance of GR- versus IR-expressing GRN signaling is under metabolic control. At the receptor level, IR60b is co-expressed with IR94f and IR94h ^17^, but it is not clear whether these IRs or yet other IRs act with IR60b in sucrose detection.

In contrast to IR60b, which is *Drosophila*-specific and restricted in expression and function, IR76b is conserved throughout insects and is broadly expressed in both olfactory and gustatory neurons of diverse chemical specificities. This broad expression is consistent with IR76b contributing to the detection of different chemicals in different neurons, with the specificity determined by the co-expressed IRs. Interestingly, IR76b is involved in the detection of polyamines by both GRNs and olfactory receptor neurons (ORNs), likely acting with different IRs in each ^9^. In antennal ORNs, IR76b is co-expressed with IR41a and together they mediate long-distance attraction to airborne polyamines, such as the evocatively named putrescine and cadaverine. However, in GRNs on the proboscis, IR76b participates in a more complex response to polyamines: female flies avoid depositing eggs on a polyamine-rich substrate unless that substrate also contains apple juice, in which case GRN detection of polyamines promotes egg-laying ^9^. This sensory integration event provides an interesting behavioral paradigm of potential ecological importance.

The precise molecular makeup of the IR complexes involved in polyamine detection is not yet clear. At the outset, the molecular composition of the polyamine receptors likely differs between GRNs and ORNs, as IR41a expression has been detected only in ORNs. In addition, neither of the two most broadly expressed co-receptors—IR25a and IR8a—is required to respond to polyamines in either context ^9^. This suggests that IR76b itself mediates the assembly of functional IR complexes. This is a surprise because, as noted above, IR76b (and IR41a) lacks the ATD domain required for the assembly and function of many iGluR family members ^2^. Furthermore, a study of *Anopheles gambiae* IRs found that ** co-expression of both AgIr76b and AgIR25a was required for AgIR41a to form ligand-gated ion channels in *Xenopus* oocytes ^21^. These data underscore the potential complexities in extrapolating from knowledge of iGluRs to IRs and even from *Drosophila* to *A. gambiae* IRs. Given the extensive sequence divergence among IRs, this is perhaps unsurprising. Nonetheless, it highlights how little is known about IRs and the need to understand how they form functional receptors.

The evolutionary relationship of IRs to glutamate receptors suggests that some IRs should respond to amino acids. This is indeed the case ^7, 22^. In the larva, IR76b is necessary for responses to amino acids ^7^. In the adult, mated females showed higher attraction to individual amino acids than virgins did, and this attraction is reported to depend on IR76b and at least one other IR, IR20a ^22^. Reminiscent of the case for polyamines, the behavioral responses to amino acids examined did not depend on IR25a or IR8a ^7, 22^, providing further support for IR76b acting as a co-receptor for more stimulus-specific IRs. Interestingly, another IR76b-dependent taste response, the low salt response in labellar taste hairs ^14^, was suppressed by the addition of IR20a ^22^. These data suggest a complex interplay between IR subunits, and possibly other factors that remain to be identified, in the formation and function of active receptor complexes.

## Ionotropic receptor “pseudo-pseudogenes” and altered specificity in the evolution of behavior

The IR gene family has undergone significant expansion and diversification among insects. Whereas some functions of the IRs seem conserved across species, as suggested by the ability of *A. gambiae* Ir76b to rescue amino-acid responses in *D. melanogaster Ir76b* mutants ^22^, in other cases, species-specific changes in IR sequences could alter ecologically relevant behaviors like preferences for specific foods or egg-laying substrates. *Drosophila sechellia*, a close relative of *D. melanogaster* (separated by only 3 to 5 million years), is a specialist that feeds exclusively on noni, the bitter and fragrant fruit of the *Morinda citrifolia* tree ^23^. This fruit is aversive and toxic for *D. melanogaster* but attractive and palatable for *D. sechellia* ^24, 25^. Recent work indicates that this dietary shift is partially due to multiple changes in the IR75 gene cluster ^26, 27^.

One initial insight to emerge from the investigation of IR75 evolution involves the molecular biology of sensory receptors. From its genomic sequence, *D. sechellia* IR75a appears to be a pseudogene because its open reading frame contains a premature stop codon. Surprisingly, this stop codon was found to undergo substantial translational read-through, allowing this “pseudo-pseudogene” to encode a functional receptor ^26^. Subsequent examination of other sensory receptors in collections of wild-caught *D. melanogaster* revealed additional examples of functional IR genes that contain premature stop codons and even an example of a GR-related olfactory receptor gene exhibiting this phenomenon ^26^. Although the molecular details of how such premature termination codon read-through occurs remain to be determined, the discovery of these “pseudo-pseudogenes” in two fly species and two sensory receptor families suggests that it is not an isolated occurrence. The implications of these findings are potentially broad. Insects, humans, and other animals contain hundreds of genes for sensory receptors (and other proteins) long presumed to be non-functional because they contain a premature stop codon. This work clearly demonstrates that such conclusions need to be revisited with functional studies. From a broader perspective, this work highlights the difficulty in extrapolating from sequence to function and emphasizes the importance of coupling sequence-based approaches to biology and evolution with experimental observation.

A second revelation from the investigation of IR75 in *D. sechellia* is the involvement of this gene cluster in altering chemical perceptions. Not only does the re-animated *IR75a* locus of *D. sechellia* encode a functional IR75a protein, but the ligand-binding domain of this receptor contains amino-acid differences that render it less sensitive to acetic acid and more sensitive to butyric acid than its *D. melanogaster* counterpart ^26^. Similarly, the ligand-binding domain of *D. sechellia* IR75b contains an amino-acid difference compared with the *D. melanogaster* ortholog that allows the *D. sechellia* receptor to respond robustly to hexanoic acid ^27^. Accompanying this shift in IR75b chemical specificity, IR75b expression is also expanded to additional olfactory neurons in *D. sechellia* compared with *D. melanogaster*, potentially increasing the salience of hexanoic acid ^27^. Together, these alterations in chemical specificity are particularly interesting as they can be related to the medium on which each species lives: hexanoic and butyric acid are abundant in the noni fruit, whereas acetic acid is common in rotting fruit, the preferred substrate of *D. melanogaster.*

Further evidence that changes in IRs may contribute to changes in preferred food and habitat comes from the observation that two IRs implicated in the detection of food-related cues in *D. melanogaster*—IR84a and IR76a—are expressed at higher levels in *D. sechellia* than in *D. melanogaster* ^28^. However, so far, there is no experimental evidence demonstrating that these regulatory differences contribute to phenotypic difference among fly species ^28^. The invasive crop pest *Drosophila suzukii*, which lays eggs in undamaged, ripening fruit, exhibits an expanded IR repertoire that could be involved in its transition from specialist to generalist ^29^. Although these studies are in their early stages, the plasticity of IR expression and function and their rapid evolutionary diversification make IRs prominent candidates for future studies of insect host range and feeding preference.

Issues of host range and feeding preference are of particular importance for disease-transmitting insects like tsetse flies ( *Glossina spp.*) and mosquitoes, which are evolutionarily separated from *D. melanogaster* by about 130 and 250 million years, respectively. As repelling or trapping disease vectors is a reasonable strategy for combatting transmission, it is important to understand the mechanisms that underlie their ability to feed, to host-seek, and to reproduce. Given the conservation of many IRs throughout dipterans and their expression in sensory tissues in both flies and mosquitoes ^1, 2, 30^, IRs are likely to play important roles in sensory transduction in insect disease vectors. Indeed, RNA interference (RNAi) knockdown of AgIR76b has been demonstrated to alter the response of mosquito larvae to butylamine ^31^.

Recent analyses have begun to examine the properties of *A. gambiae* IRs expressed in *Xenopus* oocytes. The co-expression of AgIR25a and AgIR76b with either AgIR41a or AgIR41c yielded channels that respond to amines, whereas co-expression of AgIR8a and AgIR75k conferred sensitivity to carboxylic acids ^21^. The formation of these amine- and acid-activated receptor complexes further supports similarities between IR complexes in mosquitoes and flies ^4^. As carboxylic acids synergize with other cues to promote mosquito host-seeking ^32^ and butylamine is present in human sweat ^33^, these receptors could also participate in host recognition. In addition, after female *A. gambiae* mosquitoes blood-feed, the levels of several IR RNAs in the antenna change, suggesting that this could contribute to state-dependent changes in sensory perception ^34^. The exact role of these transcript changes has yet to be analyzed.

## New roles for ionotropic receptors in sensing temperature and humidity

Recent work has also begun to extend the functions of IRs beyond chemical sensing to the detection of temperature as well as humidity ^8, 10, 12, 35, 36^. Surprisingly, IR signaling in these new contexts involves one of the main contributors to chemical sensing, the co-receptor IR25a. IR25a activity is essential for cool receptors in the larval dorsal organ as well as for moist- and dry-responsive hygro-sensory neurons in the adult antenna ^8, 10, 12, 36^. IR25a has also been implicated in the temperature-dependent resetting of the circadian clock ^35^. A second IR, IR93a, acts alongside IR25a in both thermo- and hygro-sensing, raising the possibility that IR93a acts as a thermo- and hygro-specific co-receptor with IR25a ^8, 10, 36^. As in chemical sensing, these “co-receptors” act together with other IRs specific for particular modalities: IR21a for cool sensing, IR40a for dry sensing, and IR68a for moist sensing ^8, 10, 12, 36^.

From an evolutionary perspective, these findings demonstrate that the IRs have evolved to participate in a wide range of sensory modalities, and they reveal that the IRs involved in thermo- and hygro-sensing are among the most evolutionarily conserved ^2^. IR25a, the most conserved IR, is found throughout invertebrates, whereas IR21a, IR40a, and IR93a are present in arthropods separated by more than about 650 million years of evolutionary history, and IR68a is conserved in insects separated by more than about 350 million years ^2, 15^. In contrast, most *D. melanogaster* IRs result from recent evolutionary divergence and belong to *Drosophila-*specific clades. The ancient origins of the IRs involved in thermo- and hygro-sensing suggest that these newly discovered IR functions are quite old.

Although IRs appear to operate as ligand-gated ion channels in chemical sensation, how IRs contribute to thermo- and hygro-sensation is less certain. In thermo-sensation, ectopic IR21a expression can confer cool sensitivity upon an IR25a/IR93a-expressing neuron, suggesting that this trio of IRs has a direct role in thermotransduction ^12^. Nonetheless, IRs have not yet been shown to form temperature-activated ion channels in heterologous cells, leaving open alternative mechanisms. In hygro-sensation, the situation is even more obscure, as the specific modality through which sensory neurons detect dry or moist air (thermo-sensation, mechano-sensation, chemo-sensation, and so on) is still unresolved ^37^.

## Future directions in ionotropic receptor research

IRs are now established as major contributors to odor, taste, temperature, and humidity detection in *Drosophila*. A major challenge is to understand how they perform these different roles. In olfaction and gustation, IRs appear to act as ligand-gated cation channels, but the subunit composition and stoichiometry of the receptors are unknown. It is also unclear which essential functions co-receptors like IR25a and IR8a perform and how IR76b and other IRs without traditional ATDs can serve as co-receptors or even form homomeric receptors. These challenges also apply to IR involvement in thermo-sensing and hygro-sensing, with the added issues of how the temperature and humidity detection may relate to IR-mediated chemical sensing. Do these new modalities involve significant differences in the way specific parts of the receptor, such as the ligand-binding domain, contribute to receptor gating? Are there additional critical co-factors that remain to be identified? Answers to these questions await future functional and, one anticipates, structural studies.

Beyond molecular mechanism, another open question concerns the roles of IRs in species beyond *D. melanogaster* and its close relatives. IRs are not present in humans but are conserved across arthropods. These include vectors that transmit diseases which sicken over a billion and kill over a million people annually ^38^ as well as agricultural pests estimated to destroy about 20% of global crop production ^39^. In both contexts, the ability to sense and respond to chemical, thermal, and moisture cues is central to the damage these animals cause. For example, disease-transmitting mosquitoes use these cues to help locate and feed from warm-blooded hosts ^40^. The exploration of IR function in these other species may provide important insights into how these animals execute their harmful behaviors and may identify potential molecular targets for vector and pest control. In this way, the study of IRs will have ramifications far beyond what it has already taught us about how fruit flies sense the world.
